# Limb girdle muscular dystrophy type 2B masquerading as inflammatory myopathy: case report

**DOI:** 10.1186/1546-0096-11-19

**Published:** 2013-05-03

**Authors:** Hannah Jethwa, Thomas S Jacques, Roxanna Gunny, Lucy R Wedderburn, Clarissa Pilkington, Adnan Y Manzur

**Affiliations:** 1 General Medicine, Barnet General Hospital, London, UK; 2 Neuroscience Department, Institute of Child Health, London, UK; 3 Radiology Department, Great Ormond Street Hospital, London, UK; 4 Rheumatology Unit, UCL, Institute of Child Health, London, UK; 5 Rheumatology Department, Great Ormond Street Hospital, London, UK; 6 Dubowitz Neuromuscular Centre, 30 Guilford Street, London, WC1N 1EH, UK

**Keywords:** Limb girdle muscular dystrophy, Inflammatory myositis, Monoarthritis, Paediatric

## Abstract

Limb girdle muscular dystrophy type 2B is a rare subtype of muscular dystrophy, the predominant feature of which is muscle weakness. The disease is caused by an autosomal recessively inherited reduction/absence of muscle dysferlin due to a mutation in dysferlin gene at 2p12-14. We report a 10 year old boy who presented with severe non-transient right knee pain and swelling, which later became bilateral. His pain was worst in the morning and during rest. Blood tests revealed markedly raised creatine kinase values (highest 22, 297 U/l), raising the possibility of an inflammatory myositis. MRI showed bilateral asymmetrical muscle involvement of thighs and calves with oedematous changes mimicking the imaging appearances of inflammatory myositis. CRP and ESR levels were consistently within normal limits. Over several months his knee pain worsened and limited walking. Muscle biopsy revealed a severe reduction of dysferlin immunostaining, indicating the diagnosis, which was confirmed by 2 compound heterozygous pathogenic mutations in the dysferlin gene. It is not unusual for this subtype of the disease to mimic myositis: however, significant pain is a rare presenting symptom. Given the significant overlap between this form of muscular dystrophy and inflammatory myopathies, a high index of suspicion is needed to ensure an accurate and timely diagnosis. Furthermore, characteristic inflammatory-related morning pain should not rule out consideration of non-inflammatory causes.

## Background

Muscle pain is a common presenting symptom to paediatric rheumatology clinics. The overlap between differential diagnoses can sometimes make such cases challenging. Limb girdle muscular dystrophies (LGMD) are a rare group of conditions, the predominant feature of which is weakness of lower and upper limbs, although symptoms may predominate in one muscle group. The typical presenting symptom is difficulty climbing stairs. LGMD type 2B is an autosomal recessive condition due to homozygous or compound heterozygous mutations in the dysferlin gene (DYSF) on 2p12-14, leading to a deficiency or absence of the protein, dysferlin. This protein is primarily found in skeletal muscle but can also be found in cardiac muscle [1]. The onset age of LGMD type 2 is usually 12–39 years, typically presenting with pelvic girdle weakness [2]. Pain is not a typical feature however, if present, is usually mild, transient and follows exertion. Creatine kinase (CK) levels are markedly raised and can be 10–72 times normal values [1-3]. At times, genetically inherited myopathies can mimic inflammatory myopathy. Pimentel et al. (2008) describe an adult case of LGMD type 2 subject to misdiagnosis of polymyositis, despite having consanguinous parents and also two siblings with similar symptoms of muscle weakness [4].

Although disease caused by a reduction/absence in dysferlin typically demonstrates a slow indolent course [5], in some cases symptoms may be highly debilitating [3]. Nyugen et al. (2007) describe a patient with a dysferlinopathy who had complete loss of ambulation just five years after symptom onset [5]. Given the wide spectrum of disease severity with dysferlinopathies, it is vital that healthcare professionals are aware of atypical presentations of the condition. A high index of suspicion is needed in order for an accurate diagnosis to ensure adequate and timely treatment in order to help minimize progression.

Here we report an atypical case of LGMD type 2B presenting at the age of ten years with significant, non-transient knee pain which was worst in the morning.

## Case presentation

The patient was a boy of West African origin born to non-consanguineous parents who presented aged 10 years with a one year history of bilateral knee pain, radiating down the legs. The pain started in the right knee after a long walk, was of fairly rapid onset, and was associated with swelling. Within a few weeks, his left knee subsequently also became painful and swollen. The pain was very localised to the knee and was continuous but with intermittent exacerbations, being worse in the mornings and during rest.

Non-steroidal anti-inflammatory medication prescribed by his general practitioner provided only mild symptomatic relief. Two months after symptom onset, he was reviewed by his paediatrician. Investigations demonstrated ESR and CRP values were within normal limits (5 mm/hr and 4 mg/L, respectively), as was the ACE level. He was negative for sickle cell trait. Knee MRI scan showed a small joint effusion and no synovial thickening. Aspiration and culture of synovial fluid was negative and remarkably noted as being almost acellular. Around this time a dark, ecchymosis-like rash around the affected knee developed, which was possibly secondary to warm compresses. The general paediatric team prescribed intramuscular Depo-Medrone 80mg, which only provided 5–6 days of relief. Over the following 9 months the patient was readmitted several more times with severe pain. He had no other rash, no high fevers, no eye problems other than an episode of conjunctivitis a month prior to the onset of pain. He had no ulcers, no alopecia, no profound fatigue, no soreness of the mouth, no breathlessness and no change in bowel habit.

He was an only child, born by a normal vaginal delivery at 42 weeks gestation. Both parents have sickle cell trait. There was no family history of autoimmune disease and no relevant past medical history.

On examination during his first Paediatric rheumatology appointment, he was on the 99^th^ and 98^th^ centile for his weight and height, respectively. He was afebrile with no mouth ulcers, nail fold abnormalities and no lymphadenopathy. Cardiovascular and respiratory examinations were unremarkable. He had a small right knee effusion with some pain on flexion. The knee was held in a few degrees of flexion, although it was possible to get the knee to neutral.

One month later, he was admitted to hospital following worsening of his knee pain. At this point he reported increasing difficulty standing up and needed help getting dressed and getting into the bath due to lower limb muscle weakness. On examination calf and thigh hypertrophy was noted bilaterally, the latter being more prominent. He had good strength (5/5) in his neck, upper limb, abdominal and shoulder muscles. Accurate assessment of strength was difficult because of pain and cooperation; he appeared weak in the lower limbs with 3/5 power in his quadriceps, however timed rise from floor sitting position was 1.03 seconds and timed rise from lying was 1.5 seconds (both without a Gower's manoeuvre), both of which were within the normal range.

He had tight long flexors of his arms, hamstrings and gastrocnemius, but no swelling or restriction of his joints. Hyperpigmented patches were noted over the right knee and right thigh, but no rashes were seen; again this was thought to possibly be secondary to warm compression. During neuromuscular examination right lower limb assessment was limited by significant right knee pain and he was agitated due to pain. He was able to stand on his left leg but was incapable of maintaining his balance. He had good axial flexure strength in the antigravity range. Cranial nerve examination was normal, with no facial weakness. Over the next few months his pain became progressively worse, with the pain awakening him during the night and causing him to miss days off school.

**Figure 1:**
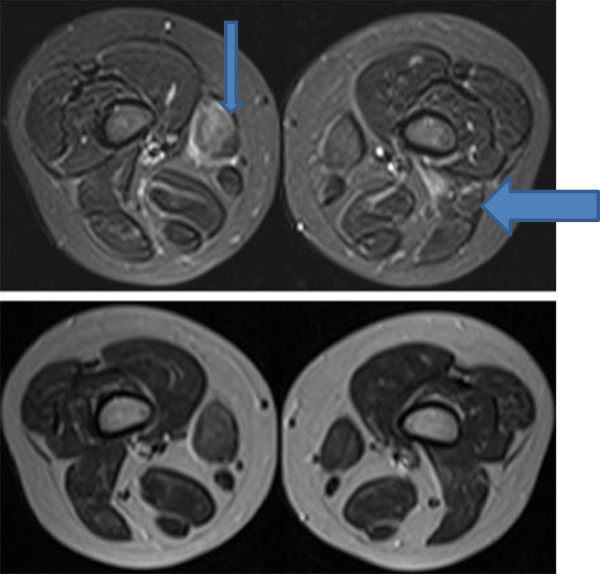
**Axial STIR (top) and T1W (below) images of the thigh muscles just above the knee showing bilateral asymmetrical muscle involvement.** The right sartorius muscle (thin arrow) shows signal hyperintensity on the STIR images and some swelling consistent with oedema. Signal hyperintensity extends just beyond the anatomical margins of the muscles into the adjacent fascia. Signal changes on the T1W images are consistent with fatty infiltration. Similar changes are seen in the insertion of the left long head of biceps femoris (thick arrow). The remainder of the posterior compartment muscles are slightly small.

**Figure 2:**
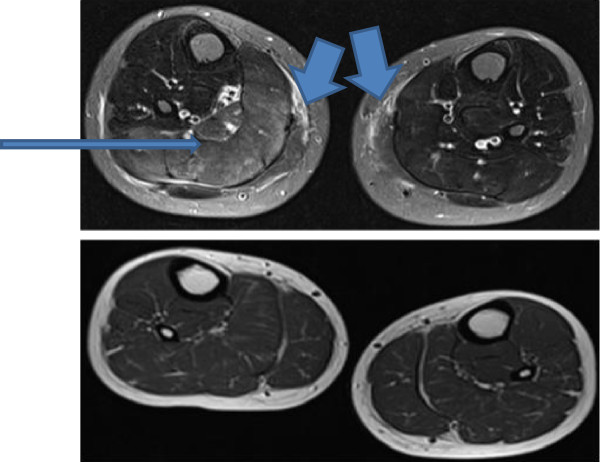
**Axial STIR (top, though with incomplete fat suppression) and axial T1W images through the calves showing various changes.** There are changes within the medial heads of both gastrocnemius muscles (short thick arrows) consistent with oedema, again with extension beyond the anatomical boundary of the muscle into the adjacent fascia. There are also some milder diffuse oedematous changes within the right soleus (thin arrow) and medial gastrocnemius muscles. No abnormal fatty changes were seen.

Blood results revealed a raised ALT (145 U/l), in the absence of liver disease. Muscle origin of this elevated transaminase was considered and serum CK was performed. CK was markedly elevated at 13,126 U/l, with an LDH 2,245 U/l. ESR and CRP were normal. ANA was negative. Muscle ultrasound scan of quadriceps femoris was normal. Leg muscle MRI revealed mildly reduced posterior compartment muscle bulk bilaterally with bilateral asymmetrical oedematous changes within selective thigh and calf muscles. Signal hyperintensity was seen on the fat suppressed STIR (short tau inversion recovery) images within the right sartorius muscle and medial heads of gastrocnemius extending beyond the anatomical boundary of the muscles into the adjacent fascia. Similar changes of muscle oedema were seen within the left biceps femoris and right soleus muscle (Figures 1 and 2). These changes are similar to the imaging appearances of inflammatory conditions such as dermatomyositis, but in an atypical distribution.

**Figure 3:**
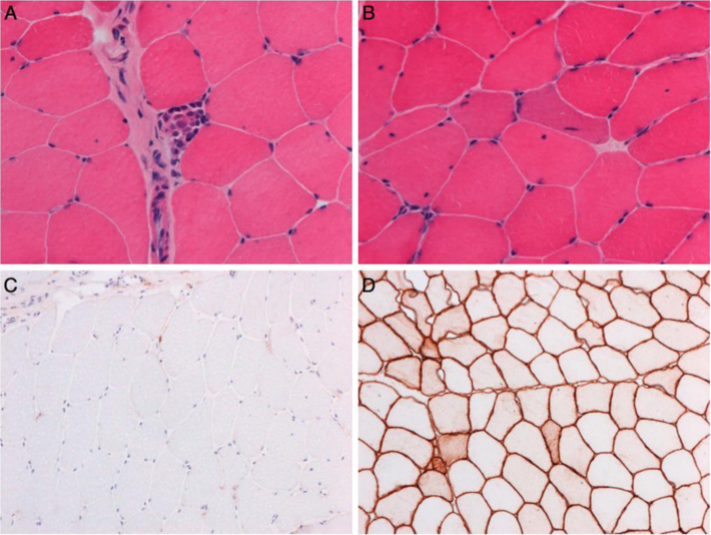
**Histology of the muscle biopsy.** Histology showed foci of necrotic fibres infiltrated by inflammatory cells (**A**, H&E); basophilic cells reminiscent of regeneration (**B**, H&E); almost complete loss of dysferlin reactivity (**C**) with maintained caveolin-3 reactivity (**D**).

Muscle biopsy revealed increased variation in fibres size (8 to 52 micrometres, approximate normal for age 31–39) due a mixture of large fibres and small rounded fibres. There were several basophilic fibres, likely to be regenerating. There was a necrotic fibre infiltrated by inflammatory cells and a few foci of perimysial and endomysial chronic inflammatory cells (Figure 3). There were a few internal nuclei but these were not excessive (<3%). There was no excess collagen deposition.

Immunohistochemistry for dysferlin was negative on most fibres. A few fibres showed focal membranous staining. Internal staining for dysferlin was not seen. An extensive panel of other dystrophy-related proteins was normal (including dystrophin, sarcoglycans, alpha-dystroglycan, merosin, emerin, collagen VI, desmin). There was some sarcolemmal staining for utrophin. There was minimal focal membrane staining for MHC class I and no staining of capillaries with Membrane Attack Complex (MAC). A few fibres expressed developmental myosin.

A provisional diagnosis of LGMD type 2B was made and was subsequently confirmed on dysferlin muscle immunoblotting and gene sequencing, which revealed two heterozygous mutations in the DYSF gene (c.265C > T; p.Arg89X in exon 4 and c.4756C > T; p.ARG1586X in exon 43). Parental DYSF studies confirmed inheritance of one of these mutations from each parent. The clinical course over a 4 month follow up period showed waxing and waning of the leg pain, with no response to a brief course of oral prednisolone treatment. The mainstay of symptom control included regular non-steroidal analgesics and management by the pain control team.

## Discussion

A central feature of the presentation in this case was muscle pain. Severe muscle pain is not typically a predominant symptom of LGMD. However, of the different types, LGMD type 2I is most closely associated with this feature [6]. Although patients with other subtypes (eg. LGMD type 2I, dystrophinopathies and facioscapulohumeral muscular dystrophy) can also present with leg pain, this is usually mild and following exertion. The aetiology of pain may be inflammatory or metabolic, and is thought to be related to hypertrophy or pseudohypertrophy of the calf muscles. Calf muscle atrophy, rather than hypertrophy, is a major feature of LGMD type 2B and pain does not typically predominate [7].

No case reports of LGMD type 2B to date describe muscle pain as a central feature. Diers et al. (2007), however, describe a patient with an unusual phenotype, with a novel compound heterozygous mutation in the Dysferlin gene and, interestingly, painful hypertrophy in calf muscles [2]. Calf swelling at onset of dysferlinopathies, however, is rare and may lead to misdiagnosis of thrombophlebitis or focal myositis [5]. Our case describes a patient with joint and muscle pain, with mild calf and thigh muscle hypertrophy. Furthermore, the pain was extremely severe, non-transient and was also present as morning pain, rather than simply pain after exertion, perplexingly suggesting an inflammatory cause. The misdiagnosis of LGMD type 2B as an inflammatory myopathy is not uncommon, especially when CK values are markedly raised [8]. This case shows MR imaging changes of muscle and perimuscular oedema similar to inflammatory conditions such as dermatomyositis. Nyugen et al. (2007) report 25% of their patients with dysferlinopathies having been misdiagnosed with polymyositis due to histological inflammatory muscle infiltrates associated with rapid progression, with or without pain [5]. A table comparing features of idiopathic inflammatory myopathies from muscular dystrophies is shown in Table 1.

**Table 1:** Comparison of idiopathic inflammatory myopathies and muscular dystrophies

	**Idiopathic inflammatory myopathies [**[9]**]**	Muscular dystrophies *
**Clinical characteristics**	Systemic symptoms and arthritis are common; rash. Systemic symptoms and arthritis are common. Onset often acute or subacute.	Selective patterns of muscle hypertrophy or atrophy, with longstanding insidious onset of motor functional difficulties
**Pattern of weakness**	Proximal, but neck flexor and abdominal weakness is a common early feature	Usually limb girdle or rarely axial or distal weakness depending on muscular dystrophy subtype
**Serum CK levels**	Typically moderately raised CK; can be normal or very high	Extremely high CK, often above 5,000 units
**Typical muscle MRI features**	Proximal muscle inflammation with high signal on T2 weighted STIR images [10]	Typically specific patterns of muscle involvement with abnormal signal on T1 weighted images in different muscular dystrophies
**Typical muscle biopsy features**	MHC – class 1 upregulation, inflammatory infiltrate (frequently peri-vascular), peri-fascicular muscle fibre atrophy and endothelial cell abnormalities [11]	Dystrophic features (fibre necrosis, regeneration, fibrosis and fat infiltration) with absence or reduction of specific protein/enzyme, depending on specific muscular dystrophy subtype.
**Genetics**	Polygenic trait	Autosomal dominant, recessive or X-linked inheritance

* Muscular dystrophies are genetically and phenotypically very heterogeneous, but with multiple subtypes; this table generalises the muscular dystrophy features.

Dysferlin deficiency on muscle immunostaining is the most pertinent feature for diagnosis of dysferlinopathies and the final confirmation is with genetic analysis of DYSF [12]. However, loss of dysferlin staining is well recognised secondary phenomena in other limb girdle muscular dystrophies, most well characterised in mutations in caveolin-3 (LGMD 1C). Sequencing of this gene is challenging as it contains 55 coding exons, spans 150kb of genomic DNA [12] and mutational hotspots do not appear to exist [12,13].

## Conclusion

Our case describes an atypical case of LGMD type 2B, with extremely elevated CK levels and severe pain and swelling of a single knee joint, and MR imaging changes initially suggestive of an inflammatory myopathy with a monoarthritis. This report supports the need for a high index of suspicion for muscular dystrophies in order to provide an early diagnosis, initiate appropriate medical care in a timely fashion and to avoid unnecessary immunosuppression for a presumed diagnosis of inflammatory myopathy.

## Consent

Written consent was obtained from the patient's mother for this publication.

## Competing interests

The authors declare that they have no competing interest.

## Authors’ contributions

HJ wrote the manuscript draft. LW, CP and AM critically reviewed drafts. TJ provided the histology data and RG reported the MRI scans. All authors read and approved the final manuscript.
